# Navβ4 regulates fast resurgent sodium currents and excitability in sensory neurons

**DOI:** 10.1186/s12990-015-0063-9

**Published:** 2015-09-25

**Authors:** Cindy Barbosa, Zhi-Yong Tan, Ruizhong Wang, Wenrui Xie, Judith A. Strong, Reesha R. Patel, Michael R. Vasko, Jun-Ming Zhang, Theodore R. Cummins

**Affiliations:** Department of Pharmacology and Toxicology, Indiana University, Indianapolis, IN USA; Medical Neuroscience Graduate Program, Indiana University School of Medicine, Indianapolis, IN USA; Department of Anesthesiology, University of Cincinnati, Cincinnati, OH USA; Department of Pharmacology and Toxicology, Stark Neurosciences Research Institute, 320 West 25th Street, NB-414F, Indianapolis, IN 46202-2266 USA

**Keywords:** Sodium channels, Resurgent currents, SCN8A, SCN4B, SCN2B, Navβ4, Nav1.6, Open-channel blocker, Sensory neurons, DRG neurons, Beta subunits, Navβ2

## Abstract

**Background:**

Increased electrical activity in peripheral sensory neurons including dorsal root ganglia (DRG) and trigeminal ganglia neurons is an important mechanism underlying pain. Voltage gated sodium channels (VGSC) contribute to the excitability of sensory neurons and are essential for the upstroke of action potentials. A unique type of VGSC current, resurgent current (INaR), generates an inward current at repolarizing voltages through an alternate mechanism of inactivation referred to as open-channel block. INaRs are proposed to enable high frequency firing and increased INaRs in sensory neurons are associated with pain pathologies. While Nav1.6 has been identified as the main carrier of fast INaR, our understanding of the mechanisms that contribute to INaR generation is limited. Specifically, the open-channel blocker in sensory neurons has not been identified. Previous studies suggest Navβ4 subunit mediates INaR in central nervous system neurons. The goal of this study was to determine whether Navβ4 regulates INaR in DRG sensory neurons.

**Results:**

Our immunocytochemistry studies show that Navβ4 expression is highly correlated with Nav1.6 expression predominantly in medium-large diameter rat DRG neurons. Navβ4 knockdown decreased endogenous fast INaR in medium-large diameter neurons as measured with whole-cell voltage clamp. Using a reduced expression system in DRG neurons, we isolated recombinant human Nav1.6 sodium currents in rat DRG neurons and found that overexpression of Navβ4 enhanced Nav1.6 INaR generation. By contrast neither overexpression of Navβ2 nor overexpression of a Navβ4-mutant, predicted to be an inactive form of Navβ4, enhanced Nav1.6 INaR generation. DRG neurons transfected with wild-type Navβ4 exhibited increased excitability with increases in both spontaneous activity and evoked activity. Thus, Navβ4 overexpression enhanced INaR and excitability, whereas knockdown or expression of mutant Navβ4 decreased INaR generation.

**Conclusion:**

INaRs are associated with inherited and acquired pain disorders. However, our ability to selectively target and study this current has been hindered due to limited understanding of how it is generated in sensory neurons. This study identified Navβ4 as an important regulator of INaR and excitability in sensory neurons. As such, Navβ4 is a potential target for the manipulation of pain sensations.

**Electronic supplementary material:**

The online version of this article (doi:10.1186/s12990-015-0063-9) contains supplementary material, which is available to authorized users.

## Background

There are several mechanisms by which pain can arise. One important mechanism is through increased firing of peripheral sensory neurons [1]. Voltage gated sodium channels (VGSC) selectively mediate the inward flow of sodium ions in response to changes in transmembrane voltage potential and generate the rapid upstroke of action potentials [2–4]. As such, VGSCs are important determinants of neuronal excitability and have been implicated in multiple pain conditions [5–9]. Sensory neurons can express an array of VGSC isoforms (Nav1s), which include tetrodotoxin sensitive (TTXS; Nav1.1, 1.2, 1.3, 1.6 and 1.7) and tetrodotoxin resistant (TTXR; Nav1.8 and Nav1.9) channels [10–12]. Human gain of function mutations in Nav1.7, and Nav1.8 are associated with painful syndromes, whereas, Nav1.7 loss of function mutations are associated with congenital insensitivity to pain (CIP) [13–24]. Interestingly, human Nav1.9 gain of function mutations have been associated with both painful neuropathy and CIP [13]. In addition, studies involving several different animal models have implicated Nav1.3 and Nav1.6 as playing potentially important roles in pain [6, 25–27].

**Fig. 1:**
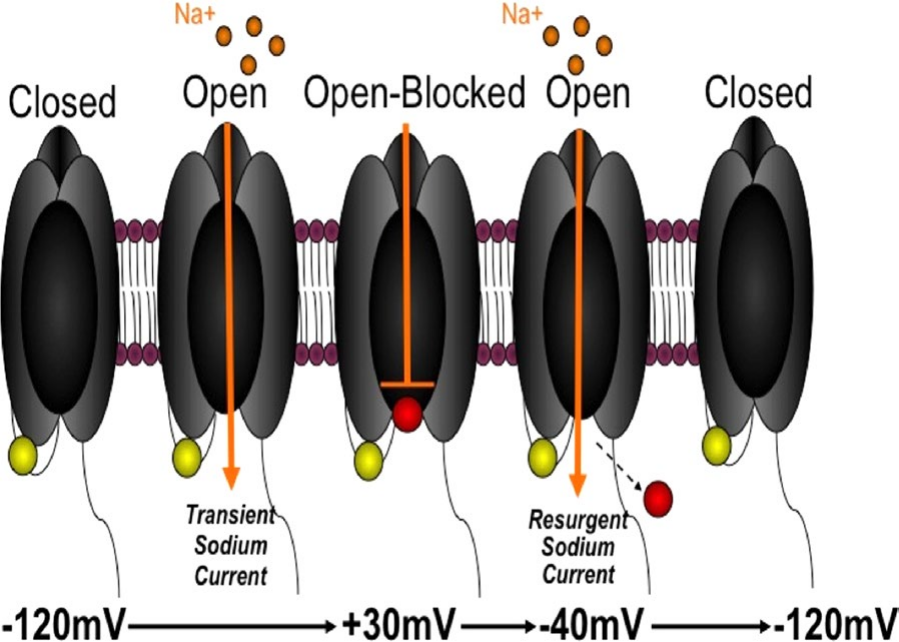
Simplified model of resurgent current generation (adapted from Cannon, 2010 [79]). Resurgent current is proposed to be generated by open-channel block mechanism. As VGSCs open upon depolarization (+30 mV), the open-channel blocker (represented as *red sphere*) competes with the intrinsic mechanism of inactivation (intracellular between DIII and DIV, represented by the *yellow sphere*). Binding of the open-channel blocker terminates transient sodium current but prevents the channel from inactivating forcing the channel to remain in an open-blocked state. Membrane repolarization (−40 mV for example) causes the blocker to unbind from the channel in the open-blocked state and resurgence of sodium conductance occurs (i.e. resurgent current). Unbinding of the blocker allows channels to return to closed state directly or as they recover from subsequent classic inactivation

Classically, VGSCs change conformation to produce channel states that are either permissive of sodium current (open state) or non-conducting (inactive and closed states). Upon depolarization of the membrane VGSCs open and quickly undergo inactivation through an intrinsic mechanism, termed fast inactivation. Once inactivated, VGSCs become refractory and require sustained hyperpolarization of the membrane before they are able to open again. An alternate mechanism for terminating transient sodium currents is through open-channel block. This mechanism underlies a unique type of voltage-gated sodium current, resurgent current [28, 29]. Although binding of the putative open-channel blocker closely mimics fast inactivation and initially prevents sodium from traversing the channel pore, unbinding allows sodium current to be uncharacteristically generated (e.g., resurge) under conditions where the channel is usually refractory. The binding of the putative open-channel blocker is voltage dependent and is thought to compete with classic fast inactivation [30–32]. The present model for resurgent current generation proposes the “blocker” binds at positive voltages (i.e. 0 to +60 mV) and subsequently unbinds during moderate (i.e. −20 to −50 mV) repolarizations (Fig. 1) resulting in inward flow of sodium current [28, 33–35]. The inward flow of resurgent sodium currents can occur during the downward phase of the action potential and may provide enough depolarizing drive to trigger subsequent action potentials, thus promoting high frequency firing [36–39].

Two types of resurgent currents have been identified in sensory neurons and are classified as slow or fast based on the kinetics of the currents. Recently, we demonstrated that slow resurgent currents are TTXR, mediated by Nav1.8 and mainly observed in a subset of small-medium diameter neurons [40]. Fast resurgent currents are TTXS and mainly mediated by Nav1.6 [41]. Under physiological conditions, fast resurgent currents are observed in a sub-population of DRG neurons (mainly medium-large diameter neurons). There is compelling evidence to support fast resurgent currents’ contribution to increased electrical activity associated with painful sensations such as paroxysmal extreme pain disorder mutations, sea-anemone toxin ATX-II induced pain and oxaliplatin acute-cooling aggravated painful neuropathy [15, 42–44]. Interestingly, knockdown of Nav1.6 (the main carrier of fast resurgent currents) blocks the development of persistent pain associated behaviors and increased activity of sensory neurons in some inflammatory and chronic pain models [25, 26]. Therefore, in normal physiology and pathophysiological states fast resurgent currents may prove to be important modulators of painful sensations.

Previous studies of fast resurgent currents in central nervous system (CNS) neurons have proposed that the open-channel blocker is part of the Navβ4 subunit [33, 35, 45]. Navβ4 is member of the family of auxiliary sodium channel subunits that associate with alpha subunits (Nav1.1-1.9), known as β-subunits [46]. To date four β-subunits (β1–β4) have been identified. β-subunits can associate with alpha-subunits non-covalently (β1, β3) or covalently (β2, β4) and may modulate VGSC gating, assembly and localization [46, 47]. These subunits share some common features: a single transmembrane domain, a cytoplasmic C-terminal domain, and an extracellular N-terminal domain. However, only the Navβ4 subunit has a cytoplasmic tail with a region of positively charged and hydrophobic residues that is predicted to have the necessary properties to act as the voltage dependent open-channel blocker [35]. Based on this observation, a synthetic β4 peptide has been designed from the cytoplasmic tail of the Navβ4 [35, 48]. The synthetic β4-peptide can reconstitute resurgent currents in cerebellar granule neurons and other cell lines that do not exhibit endogenous resurgent current. However, the role of Navβ4 is not fully understood since heterologous expression of full length Navβ4 is not sufficient to recapitulate resurgent current generation [33, 44, 49–51].

Our knowledge of the molecular determinants of fast resurgent currents in sensory neurons is limited. In order to address the current gap in our understanding, we investigated the identity of the potential open-channel blocker that mediates fast resurgent currents. Because Navβ4 is expressed in DRG sensory neurons and has the potential to act as the open-channel blocker [46, 52], we investigated if Navβ4 functionally regulates fast resurgent current generation in DRG neurons. While slow resurgent currents are distinct in several aspects, it is possible that our findings may be applicable to generation of this current as well.

Our results show that Navβ4 regulates fast resurgent currents and excitability in sensory neurons. Navβ4 expression is correlated with Nav1.6 (the main carrier of fast resurgent current) in DRG neurons [41, 53, 54]. Furthermore, Navβ4 knockdown decreased resurgent currents, whereas, wild-type Navβ4 (Navβ4-WT) overexpression enhanced resurgent currents and excitability. The C-terminus of Navβ4-WT is important for enhancement of resurgent current as a Navβ4 construct with key C-terminal mutations was unable to enhance resurgent currents, supporting the model of Navβ4-WT C-terminus mediating open-channel block. As such, Navβ4 presents a potential target for the study and treatment of pain.

## Results

### Navβ4 and Nav1.6 expression are correlated in DRG neurons

**Fig. 2:**
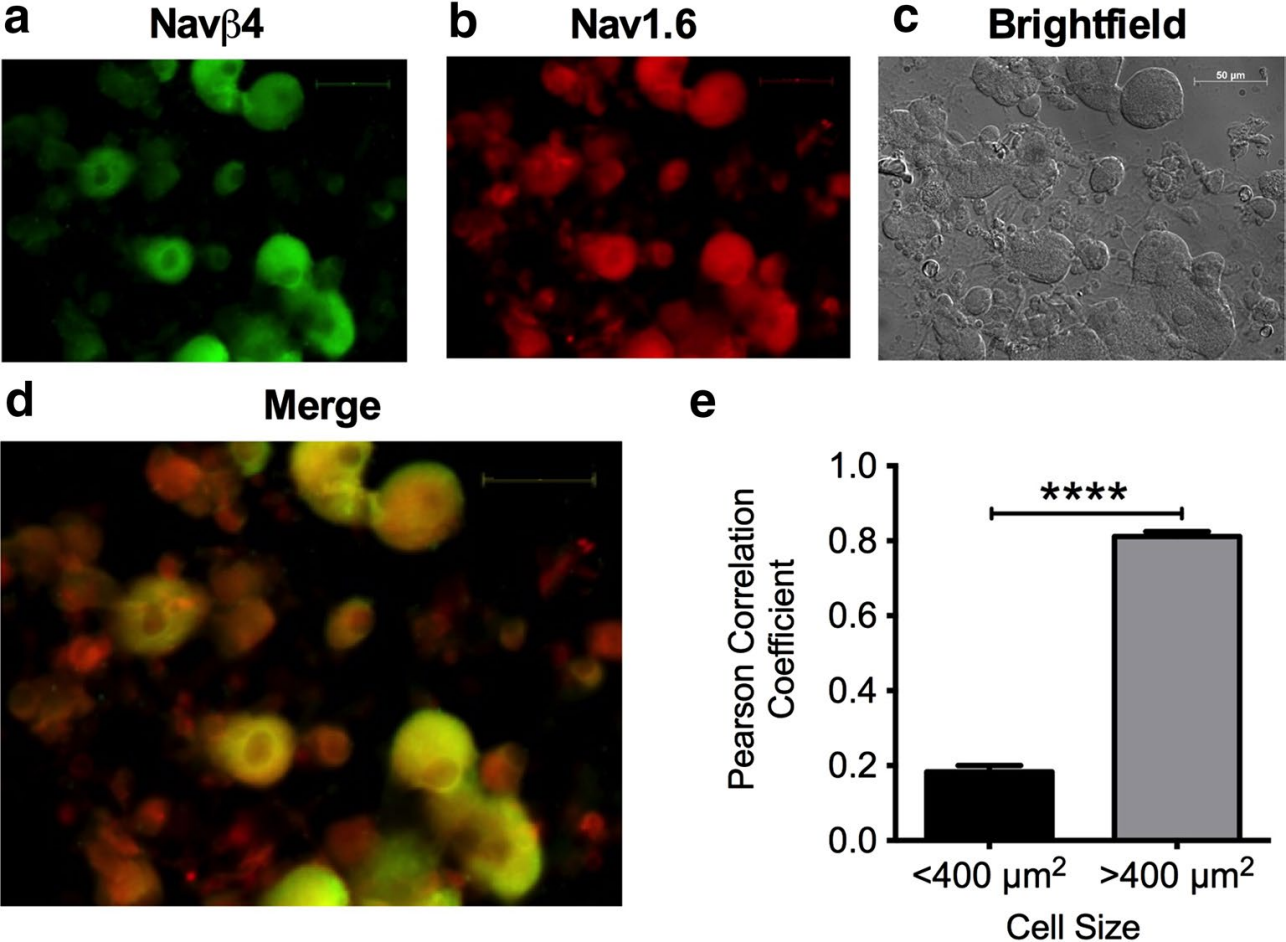
Expression of Nav1.6 and Navβ4 in DRG neurons. Examples of immunocytochemical staining of Navβ4 and Nav1.6 in primary cultured DRG neurons are shown in **a** and **b**. Navβ4 signal is shown in *green* and Nav1.6 signal is shown in *red* with corresponding brightfield (DIC) image shown in **c**. Merged image (**d**) shows that some DRG neurons but not all express both Nav1.6 and Navβ4. **e** Strong co-expression of Nav1.6 and Navβ4 signal is mainly observed in medium-large diameter neurons (>400 um^2^, n = 344), but not small diameter neurons (<400 um^2^, n = 347) as indicated by the Pearson correlation coefficient. *Asterisks* (****) represent p < 0.0001 obtained from student’s t test. *Scale bar* 50 μm

The goal of our study was to determine if Navβ4 regulates fast resurgent currents in DRG neurons. Therefore, we examined the expression pattern of Navβ4 in rat DRG neurons and its correlation with Nav1.6. Immunostaining of DRG neurons in dissociated cultures shows that Navβ4 signal is present in all size classes. However, mean intensity of staining was threefold higher in medium-large diameter neurons than small diameter neurons (mean intensity p < 0.0001: medium-large diameter, 49.5 ± 2 A.U., n = 260; small diameter 17.4 ± 0.5 A.U., n = 557). These medium-large diameter neurons, with an estimated cross sectional area >400 µm^2^, most likely give rise to Aδ and Aβ fibers. Our results are consistent with previous studies, which found Navβ4 mRNA levels to be higher in medium-large diameter neurons relative to small diameter neurons [42, 55]. Similarly Nav1.6 signal is observed in all size classes but exhibits more pronounced immunostaining in medium-large diameter neurons, consistent with previous observations [6, 56]. Mean intensity of staining was twofold higher in medium-large diameter neurons than small diameter neurons (mean intensity p < 0.0001: medium-large diameter, 62.5 ± 2 A.U., n = 260; small diameter 26.94 ± 1 A.U., n = 557). Representative images are shown in Fig. 2a–c. Co-expression of the two signals (Navβ4-green and Nav1.6-red) in neurons was quantified in a separate analysis using the Pearson correlation coefficient method. Using this approach, we found that medium-large diameter neurons have a stronger correlation between Nav1.6 and Navβ4 expression than small diameter neurons (Fig. 2d, e, Pearson correlation coefficient p < 0.0001: medium-large diameter, 0.81 ± 0.01, n = 344; small diameter, 0.18 ± 0.02, n = 348).

### Navβ4 knockdown reduces resurgent current generation

**Fig. 3:**
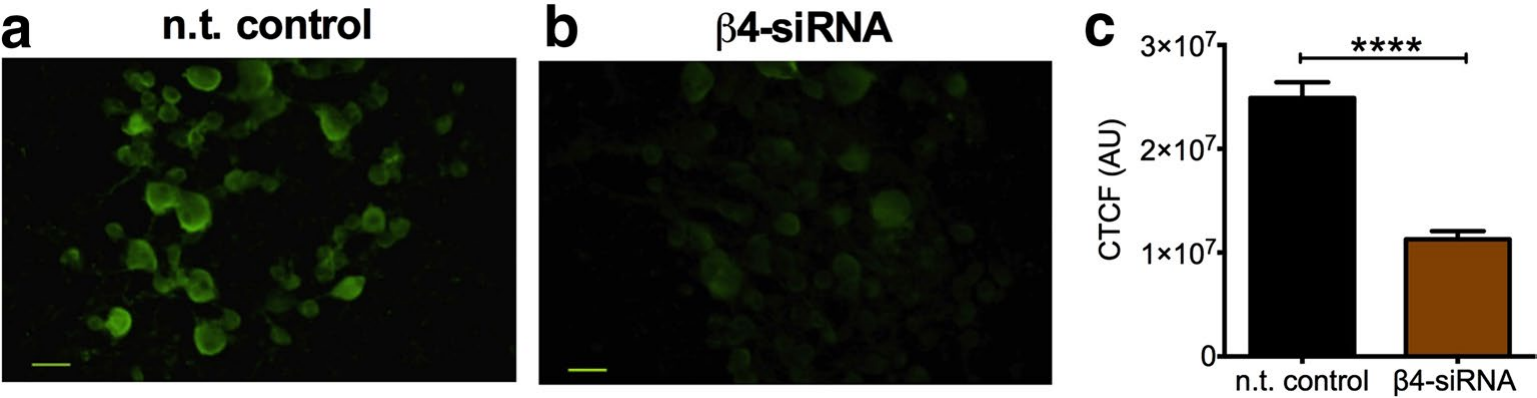
In vivo knockdown decreases Navβ4 expression levels. Representative images of immunocytochemical staining of Navβ4 in primary cultured DRG neurons from rats injected with non-targeting siRNA (n.t. control, **a**) or Navβ4 siRNA (β4siRNA, **b**) 72 h post injection. **c** Corrected total cell fluorescence (CTCF) is significantly decreased in DRG neurons from rats injected with β4siRNA (n = 352 from three rats, p < 0.0001) compared with n.t. control (n = 264 from three rats). *Scale bar* 50 μm. *Asterisks* (****) represent p < 0.0001 obtained from student’s t test

We next investigated if Navβ4 functionally regulates endogenous fast resurgent current generation in DRG neurons. In order to address this question we used an in vivo knockdown approach [57]. Animals received localized injections (near L4 and L5 DRG) of Navβ4 siRNAs (Navβ4siRNA) or non-targeting control siRNAs (n.t. control) mixed with the transfection reagent JetPEI (see “Methods” section). Three days post injection DRG neurons were cultured. After 16–36 h in culture, DRG neurons were examined by immunocytochemistry to verify knockdown and subjected to whole-cell patch-clamp recordings to assess sodium current properties. Representative images of Navβ4 staining in n.t. control and Navβ4siRNA groups are shown in Fig. 3a, b. Navβ4 levels were reduced in the Navβ4siRNA group by approximately 50 % compared to n.t. control (Fig. 3c, mean corrected total cell fluorescence difference p < 0.0001: n.t. control group, 1.3 ± 0.08 × 10^7^ A.U, n = 352; β4siRNA group, 2.5 ± 0.15 × 10^7^ A.U., n = 264).

**Fig. 4:**
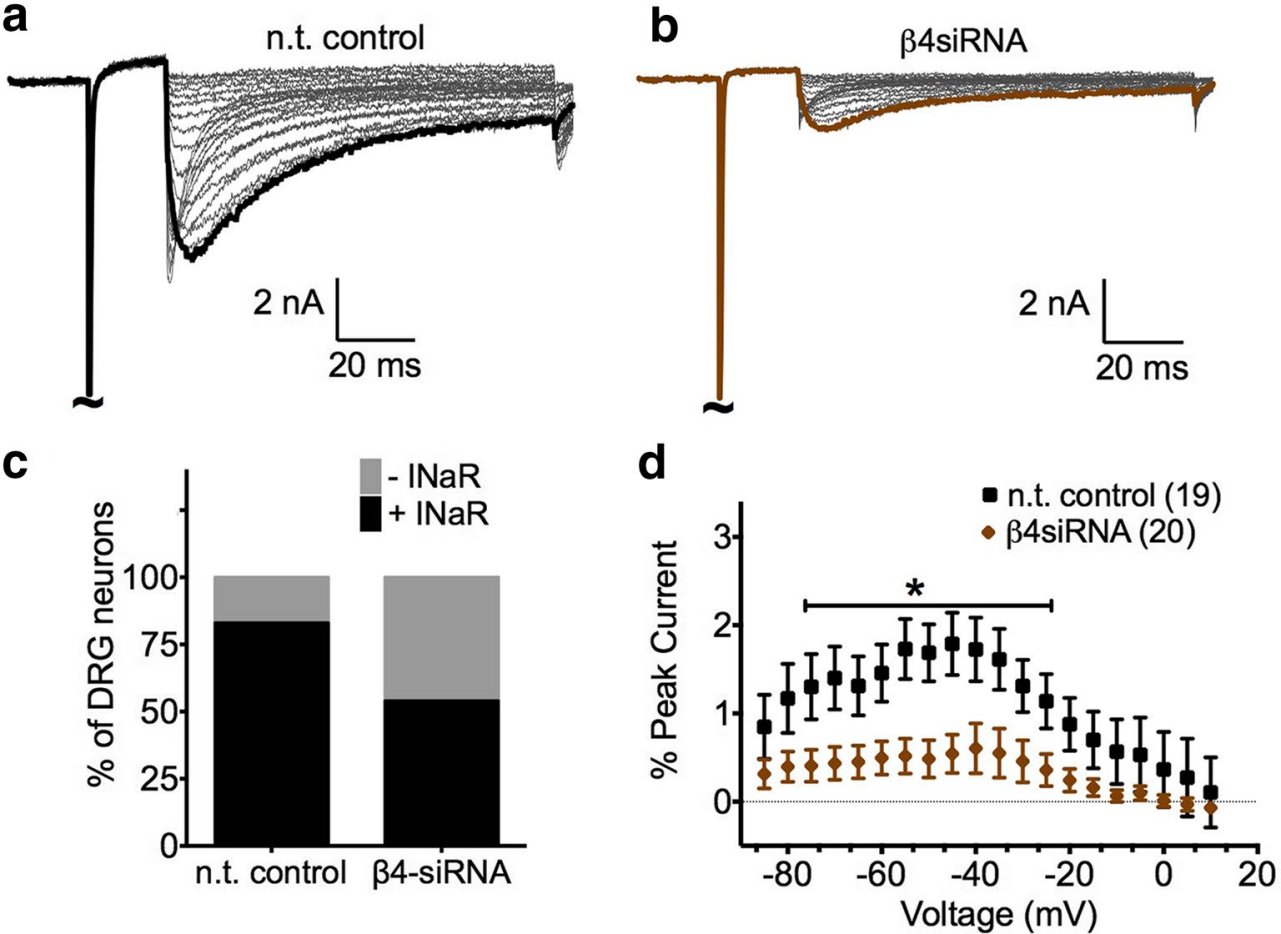
Navβ4 knockdown reduces endogenous fast resurgent current. Representative traces of endogenous fast resurgent currents obtained from medium-large diameter DRG neurons cultured from rats injected with n.t. control (**a**) or β4siRNA (**b**). Peak resurgent current traces are highlighted with *black* (n.t. control) and *brown* (β4siRNA). **c** Compared with n.t. control, β4siRNA significantly decreased the percentage of DRG neurons that generated resurgent currents (p < 0.0005, χ^2^ test). **d** Compared to control (*black squares*, n = 19), Navβ4 knockdown (*brown diamonds*, n = 20) significantly decreased resurgent current amplitude in a range of voltages. (*p < 0.05 obtained from Student’s t test). Note that resurgent currents were normalized to peak transient currents and plotted as a function of voltage. Summary data are mean ± SEM

Endogenous sodium currents were recorded from medium-large diameter DRG neurons cultured from rats treated with Navβ4siRNA or n.t. control. Small diameter neurons were excluded because these neurons do not endogenously generate fast resurgent current under normal conditions [41, 42], possibly reflecting the weak correlation of Nav1.6 and Navβ4 expression (Fig. 2). Cell size was assessed by measurement of total membrane capacitance. In the 39 cells examined, the average cell capacitance was 60.2 ± 2.9 pF; cell size was not significantly different between n.t. control and Navβ4siRNA groups. The peak current density of transient currents, which can contain both fast (TTXS) and slow (TTXR) sodium currents, was not significantly different. However, when the TTXS component was isolated by prepulse subtraction [58], there was a 32 % reduction in the peak current density with Navβ4 siRNA treatment (Additional file 1: Figure S1). In order to determine relative changes in fast resurgent currents (which are TTXS) and exclude potential effects due to changes in TTXS channel density, resurgent current amplitudes were normalized to TTXS peak transient current amplitudes. Resurgent currents were examined using a two-step pulse protocol. First, the cells were conditioned to +30 mV for 20 ms followed by 100 ms repolarization pulses ranging from +15 to −85 mV (in 5 mV increments) to test for resurgent current. Figure 4a, b, show representative traces of endogenous fast resurgent current obtained from each group. Navβ4 siRNA treatment significantly reduced the fraction of fast resurgent current positive neurons relative to n.t. control (Fig. 4c, χ^2^ test, p < 0.05). In the Navβ4siRNA group, 7 out of 20 neurons (35 %) generated fast resurgent current, whereas in the n.t. control group, 15 out of 19 (79 %) neurons were fast resurgent current positive. Moreover, average fast resurgent current amplitude (expressed as a percentage of peak transient TTXS current) was significantly decreased in the Navβ4siRNA group compared to control (Fig. 4d, n.t. control 1.79 ± 0.36 %, n = 19; β4siRNA 0.60 ± 0.28 %, n = 20).

Navβ4 may also alter the voltage-dependence of activation and inactivation of VGSCs [59]. For example, in cerebellar granule neurons Navβ4 knockdown shifted the voltage-dependence of inactivation to more negative potentials, whereas, activation was unchanged [33]. In contrast, in heterologous expression systems, Navβ4 co-expression shifts the voltage-dependence of activation to negative potentials but no change in inactivation is observed [46, 49, 60]. Modulation by Navβ4 seems to be cell background specific. In the endogenous sodium current recordings we obtained, activation was not studied due to contamination with TTXR currents that cannot be readily subtracted. However, using pre-pulse subtraction, we isolated the TTXS component of the sodium currents recorded with the steady-state inactivation protocol and compared the voltage-dependence of inactivation between groups (see “Methods” section). Navβ4 knockdown slightly shifted the voltage dependence of inactivation to more hyperpolarized potentials. This apparent shift did not quite reach significance (Additional file 1: Fig S1. midpoint of inactivation, p = 0.052; n.t. control −67.7 ± 2.4 mV, n = 18; β4siRNA −73.6 ± 2.6 mV, n = 20).

### Navβ4 increases Nav1.6r resurgent currents whereas Navβ2 does not

Using a reduced system we studied the effects of overexpression of Navβ4 and Navβ2 on Nav1.6 mediated fast resurgent currents. Navβ4 is most homologous to Navβ2, with 35 % identity [46]. While they are structurally similar, Navβ2 has been proposed to lack the appropriate properties to enable open-channel block [35, 48]. Therefore, we hypothesized that Navβ4 overexpression would increase fast resurgent current, whereas Navβ2 would not. Interestingly, both subunits contain a free cysteine that is likely to form a disulfide bond with the α-subunit [46, 61, 62]. While it is not known if Navβ2 and Navβ4 compete for the same cysteine on the α-subunit, if they do compete, then Navβ2 would be predicted to decrease fast resurgent current.

**Fig. 5:**
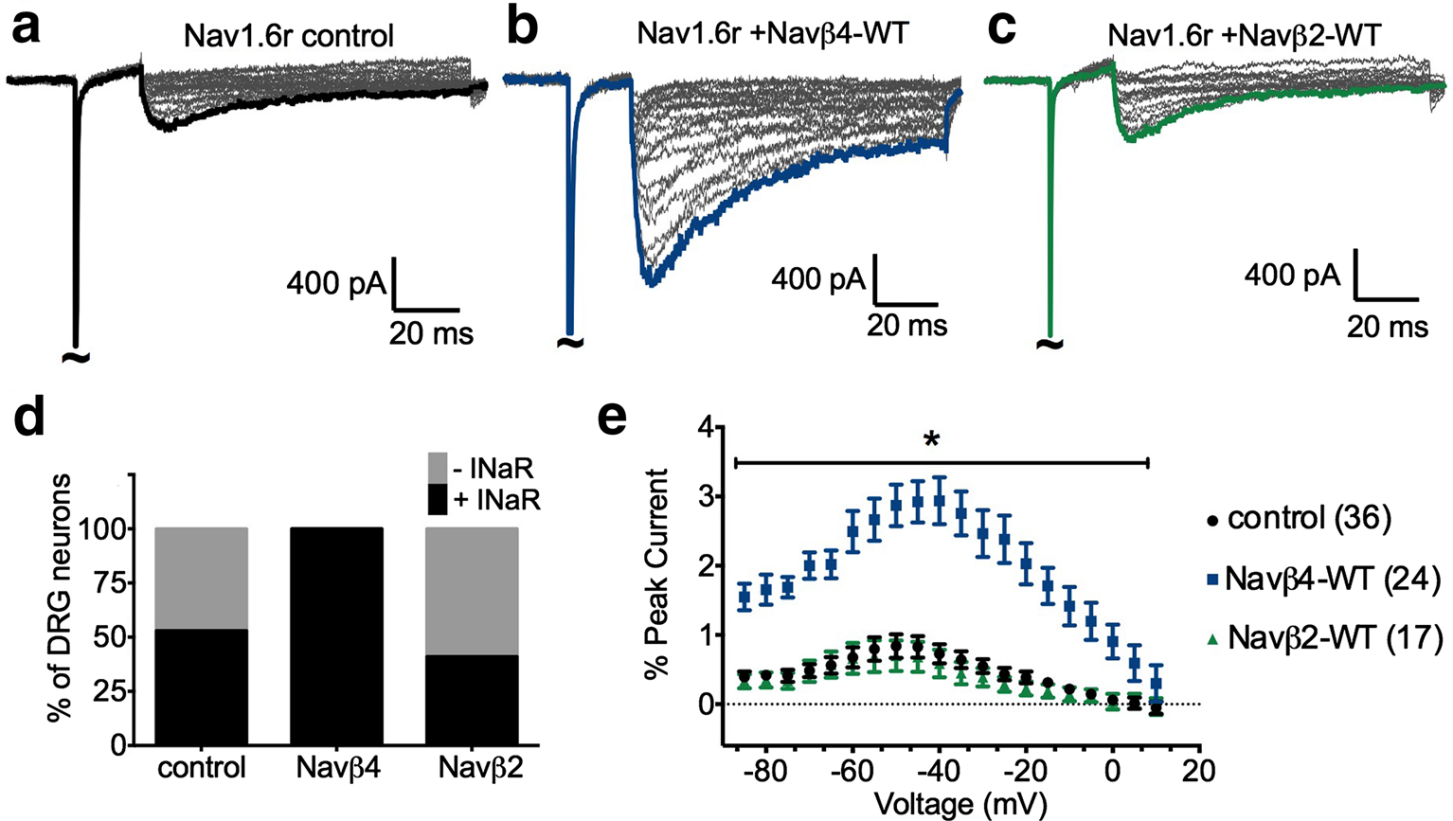
Overexpression of Navβ4-WT of DRG neuron increases Nav1.6r resurgent currents whereas Navβ2-WT does not. DRG neurons were transfected with Nav1.6r and Navβ4-WT, Navβ2-WT or fluorescent protein tag (control). Beta subunits were tagged with fluorescent protein to monitor expression. Representative traces were obtained from transfected DRG neurons and peak resurgent currents are highlighted for control (**a** *black*), Navβ4-WT (**b** *blue*), and Navβ2-WT(**c** *green*). **d** Overexpression of Navβ4-WT significantly increased the percentage of DRG neurons that generated resurgent current compared to control (p < 0.0001, χ^2^ test). The percentage of DRG neurons that generated resurgent currents was not different between Navβ2-WT and control groups. **e** Compared to control (*black circles*, n = 36), overexpression of Navβ4-WT (*blue squares*, n = 24) increased resurgent current amplitude. Navβ2-WT (*green triangles*, n = 17) did not alter resurgent current amplitude relative to control. Note that resurgent currents were normalized to peak transient currents and plotted as a function of voltage. Summary data are mean ± SEM

Dissociated DRG neuronal cultures were co-transfected with recombinant human Nav1.6 and either wild-type (WT) Navβ4 or Navβ2. We used a Nav1.6 construct (Nav1.6r) that has been mutated to be resistant TTX [63]. This allows 500 nM TTX to be used to block endogenous TTXS currents without blocking Nav1.6r currents, enabling pharmacological isolation of Nav1.6r mediated fast resurgent currents [15] (see “Methods” section). β-Subunit constructs were tagged with a fluorescent protein (Venus or Turquoise) at the C-terminus to verify expression. As a control, Nav1.6r was co-expressed with fluorescent protein (corresponding to the tag) by itself. In addition, Nav1.8 was knocked down with cotransfection of a Nav1.8 shRNA-IRES-dsRED construct to minimize contamination by Nav1.8 currents [15, 64] (see “Methods” section). Whole-cell patch-clamp recordings were obtained 2–3.5 days post-transfection. Representative traces of Nav1.6r resurgent currents recorded with co-transfection of control (fluorescent tag), Navβ4-WT and Navβ2-WT are shown in Fig. 5a–c. The percentage of transfected cells exhibiting fast resurgent current increased significantly with Navβ4-WT overexpression (Fig. 5d, χ^2^ test, p < 0.0001). For Navβ4-WT group, 24 out 24 cells (100 %) generated fast resurgent current, whereas in control 20 out of 36 cells (56 %) exhibited identifiable fast resurgent currents. No difference was observed between Navβ2 resurgent current frequency (7 out of 17 cells, 41 %) and control. Overexpression of Navβ4-WT also resulted in a threefold increase in fast resurgent current amplitude relative to control (Fig. 5e, p < 0.0001; control 0.84 ± 0.2 %, n = 36; Navβ4-WT 2.94 ± 0.3 %, n = 24). In contrast, there was no difference in fast resurgent current amplitude between the Navβ2-WT group and control (0.7 ± 0.2, n = 17).

Nav1.6r transient current recordings were analyzed for potential alterations in activation, steady-state fast inactivation and current density. Overexpression of Navβ4-WT caused a significant decrease in current density, a depolarizing shift in inactivation and no change in activation relative to control. No significant changes in any of the parameters studied where observed with Navβ2-WT overexpression relative to control (Additional file 2: Figure S2; Table 1).

**Table 1:** Parameters of human Nav1.6r transient currents with beta subunit co-expression

	Activation		Inactivation		Current density (nA/pF)
	V_1/2_ (mV)	k	V_1/2_ (mV)	k	
Control n	−41.1 ± 1.7 34	5.3 ± 0.3 34	−76.2 ± 1.3 35	6.2 ± 0.1 35	2.1 ± 0.4 36
Navβ2 n	−46.0 ± 2.5 15	4.2 ± 0.3* 15	−77.1 ± 2.5 15	7.2 ± 0.2^#^ 15	2.3 ± 0.4 15
Navβ4-WT n	−42.4 ± 1.8 24	5.9 ± 0.4 24	−72.3 ± 1.8* 21	6.0 ± 0.1 21	1.0 ± 0.2* 24
Navβ4-Mt n	−30.5 ± 1.9^#,†^ 16	5.7 ± 0.3 16	−67.1 ± 1.7^#,†^ 16	8.3 ± 0.5^#,†^ 16	1.3 ± 0.3 16

Groups were compared to control using Student t-test. Data are mean ± SEM

*k* slope factor of voltage dependence of (in) activation, *V*_*1*/*2*_ voltage of half maximal (in) activation

* P < 0.05 (vs control); ^#^P < 0.0005 (vs control); ^†^ P < 0.0005 (vs Navβ4)

### Navβ4 C-terminus is important for mediating Nav1.6r resurgent currents

**Fig. 6:**
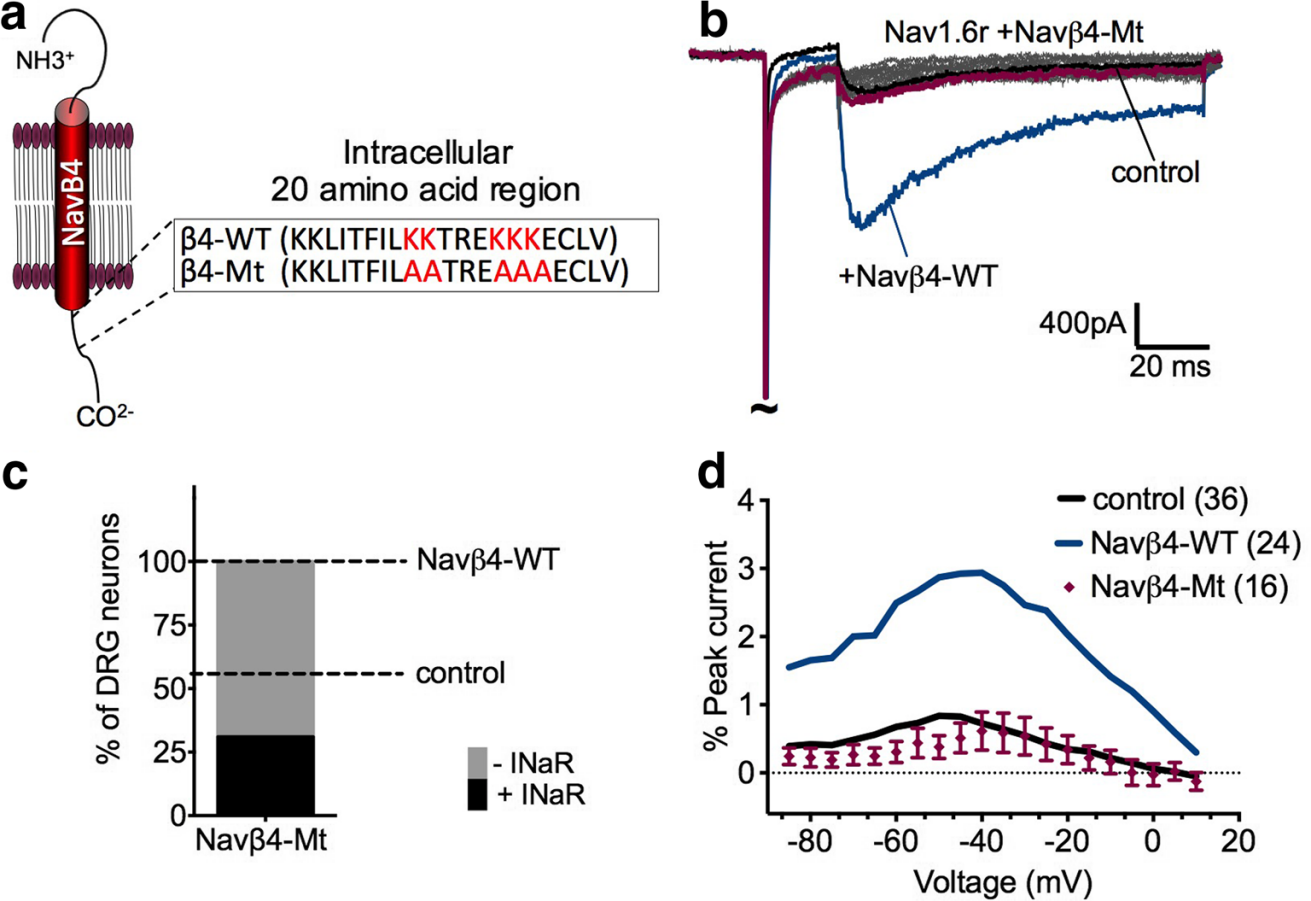
Navβ4 C-terminus is important for positive regulation of resurgent currents. **a** Illustration of Navβ4 subunit, which consists of an extracellular immunoglobulin-like domain, a single transmembrane domain, and a cytoplasmic tail. The cytoplasmic tail contains the β4 peptide sequence (amino acids 183–203) proposed to mediate resurgent currents. *Inset* highlights the 20 amino acids segment sequence of the cytoplasmic tail of Na_V_β4 corresponding to residues 183–203 of the rat protein. *Red lettering* indicates five lysine residues corresponding to Navβ4-WT (Β4-WT) sequence in C-terminal region that were neutralized to alanine to generate a predicted inactive form of Navβ4 (Navβ4-Mt). **b** Representative trace obtained from transfected DRG neurons with Nav1.6r and Navβ4-Mt. Navβ4-Mt peak resurgent current is highlighted in *purple*. For comparison representative trace of peak resurgent current obtained from Navβ4-WT group is highlighted in *blue* and control is highlighted in *black*. **c** Overexpression of Navβ4-Mt significantly decreased the percentage of DRG neurons that generated resurgent current compared to Navβ4-WT (p < 0.0001, χ^2^ test) but not significantly different to control. **d** Resurgent current amplitude in Navβ4-Mt group (*purple circles*, n = 16) was significantly decreased compared to Navβ4-WT (*blue line*) but not different to control. Note that resurgent currents were normalized to peak transient currents and plotted as a function of voltage. Navβ4-WT and control data is plotted as a line of the average for reference. Navβ4-Mt summary data are mean ± SEM

We next investigated if the C-terminal region proposed to act as an open-channel blocker (amino acids: 184–203; β4 peptide) [33, 35, 44] was important for Navβ4-WT positive regulation of fast resurgent currents in DRG neurons. While the β4-peptide can recapitulate resurgent currents in various cell types [48], the role of this region has not been studied in full length Navβ4. Therefore, we generated a mutant Navβ4 (Navβ4-Mt) in which five lysine residues at positions 192–193 and 197–199 were converted to alanine (Fig. 6a). These residues were chosen because the positive charges at these positions are highly conserved and have been shown to be important for β4-peptide open-channel blocker activity [35, 48]. Based on peptide studies we predicted that the C-terminus of Navβ4-Mt would not be able to bind to the sodium channel pore and therefore would not facilitate fast resurgent current generation.

In contrast to Navβ4-WT, overexpression of Navβ4-Mt did not enhance fast resurgent current generation. Figure 6b, shows a comparison of representative traces of Nav1.6r resurgent currents recorded after co-transfection of Navβ4-Mt, control (fluorescent tag) and Navβ4-WT. Overexpression of Navβ4-Mt decreased the frequency of fast resurgent current positive neurons relative to neurons transfected with Navβ4-WT (Fig. 6c: % of fast resurgent current positive neurons, Navβ4-Mt, 31 %, χ^2^-test, p < 0.0001) but was not significantly different from control (p = 0.053). Similarly, fast resurgent current amplitude was significantly decreased relative to Navβ4-WT (Fig. 6d, p < 0.0001; Navβ4-Mt 0.61 ± 0.3 %, n = 16) but was not significantly different compared to control. Analysis of transient current recordings revealed that Navβ4-Mt overexpression shifted the voltage dependence of steady-state fast inactivation and activation to positive potentials relative to Navβ4-WT and control (Additional file 2: Figure S2; Table 1).

### Navβ4 expression increases excitability of DRG sensory neurons

**Fig. 7:**
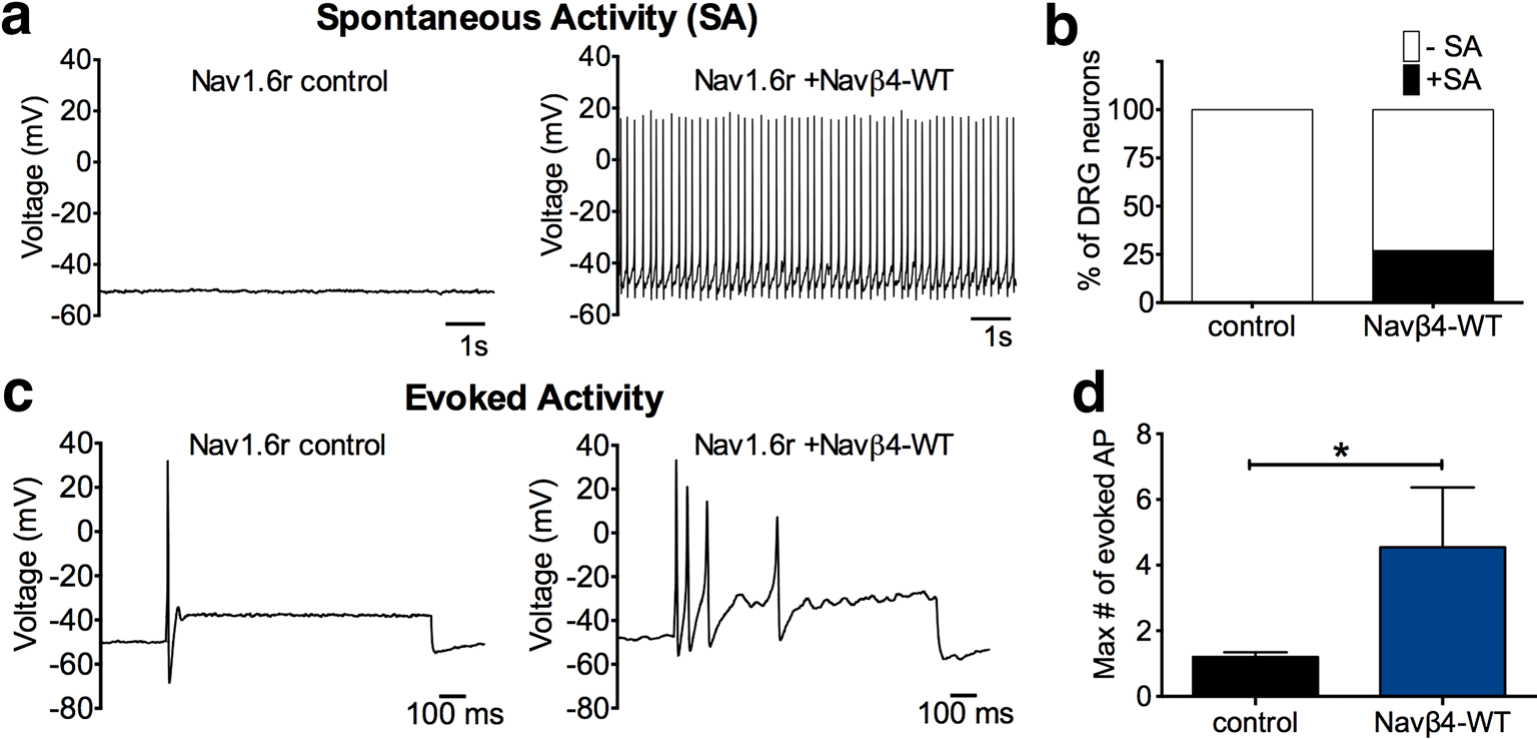
Overexpression of Navβ4-WT increases excitability of DRG neurons. DRG neurons co-transfected with Nav1.6r and fluorescent probe tag (control) or Navβ4-WT were examined under current clamp conditions. 500 nM TTX was included in the bath solution to block endogenous TTXS channels and Nav1.8 was knocked-down with shRNA. **a** Representative traces of spontaneous activity recorded in control (*left panel*) and Navβ4 (*right panel*) groups. **b** Overexpression of Navβ4 (n = 14) increased the percentage of neurons that were spontaneously active (p < 0.05, Fisher’s exact test) compared to control (n = 15). Cells that were not spontaneously active were examined for evoked activity. A series of 1 s depolarizing current steps (0 pA up 1.2 nA in 100 pA increments) were injected into transfected DRG neurons from their resting membrane potentials. **c** Representative membrane responses to current injections at rheobase in control (*left panel*) and Navβ4-WT (*right panel*). **d** Compared with control, Navβ4-WT overexpression significantly increased the maximum number of evoked action potentials. The maximum number of evoked action potentials was defined as the maximum number of action potentials elicited at a given step depolarization from 0 pA to 1.2 nA for each cell. Data are mean ± SEM. *p < 0.05, Student’s t test

Our data demonstrate that Navβ4-WT overexpression increased Nav1.6r resurgent currents in DRG neurons. Therefore, we used this as an opportunity to study the impact of increased fast resurgent current on DRG neuronal excitability. DRG neurons were co-transfected with Nav1.6r and either the fluorescent tag (control) or Navβ4-WT. Current clamp experiments were conducted on transfected neurons in which Nav1.6r sodium currents were isolated as previously described using Nav1.8 shRNA and pharmacological tools. Figure 7a shows a representative trace of spontaneous activity recorded in the Navβ4-WT group; spontaneous action potential activity was not observed in control neurons. Four out of 15 cells (27 %) transfected with Navβ4-WT were spontaneously active while zero of 15 control cells (0 %) were spontaneously active. Thus, overexpression of Navβ4-WT significantly increased spontaneous activity (Fig. 7b: χ^2^ test, p < 0.05). In cells that did not exhibit spontaneous activity, neuronal excitability was examined by a series of 1 s depolarizing current injections (0 pA up to 1.2 nA in 100 pA increments) from their resting membrane potentials. Representative membrane responses to current injections (evoked activity at rheobase) in control and Navβ4-WT transfected neurons are shown in Fig. 7c. Navβ4 overexpression significantly increased the maximum number of evoked action potentials (Fig. 7d: Navβ4-WT 4.5 ± 1.8, n = 11; control 1.2 ± 0.1, n = 15, p < 0.05). The maximum number of evoked action potential was defined as the maximum number of action potentials elicited at a given step depolarization from 0 pA to 1.2 nA for each cell. The number of evoked action potentials was also significantly greater with Navβ4 overexpression when measured specifically at rheobase, and at 2× and 3× rheobase compared to control (Additional file 3: Figure S3). In cells that we examined for evoked activity no significant change was observed in resting membrane potential, input resistance or rheobase (Table 2).

**Table 2:** Excitability parameters measured of non-spontaneously active cells in control and Navβ4-WT groups

Parameters	Control	Navβ4
Resting membrane potential (mV) n	−47.3 ± 1.8 15	−48.8 ± 2.4 11
Input resistance (MΩ) n	236 ± 40 15	345 ± 62 11
Rheobase (pA) n	287 ± 37 15	227 ± 38 11

No significant difference were observed when groups were compared using Student’s t test

## Discussion

In this study we tested the hypothesis that Navβ4 is critical to the generation of fast resurgent sodium currents in DRG sensory neurons. Five principle findings support the conclusion that Navβ4 is the primary open-channel blocker underlying sensory neuron fast resurgent currents. First, Navβ4 antibody staining is highly correlated with Nav1.6 antibody staining, and this relationship is predominantly observed in medium-large neurons where endogenous fast resurgent currents are typically observed. Second, Navβ4 knockdown substantially attenuates endogenous fast resurgent sodium currents in medium-large diameter sensory neurons. Third, co-expressing Navβ4 with recombinant Nav1.6r channels in cultured sensory neurons resulted in a threefold increase in Nav1.6r resurgent current amplitude relative to control. Fourth, co-expressing the closely related Navβ2 with recombinant Nav1.6r channels in cultured sensory neurons did not increase Nav1.6r resurgent current amplitude relative to control. Fifth, co-expressing a mutant Navβ4 with recombinant Nav1.6r channels in cultured sensory neurons also did not increase Nav1.6r resurgent current amplitude relative to control, although other channel properties were altered. In addition, co-expressing the wild-type Navβ4 with recombinant Nav1.6r channels in cultured sensory neurons substantially increased neuronal excitability, indicating that resurgent sodium currents are important regulators of sensory neuron action potential activity.

Sensory neurons express multiple voltage-gated sodium channel isoforms, including TTXS and TTXR channels. In a previous study, fast resurgent sodium currents were observed in 44 % of mouse sensory neurons with larger diameter somas, but were not observed in neurons from Nav1.6-knockout mice [41]. In the current study we observed a high degree of association between Nav1.6 and Navβ4 antibody staining in medium-large diameter neurons, 79 % of which generated fast resurgent currents. Although the immunocytochemistry we performed demonstrates that expression of Nav1.6 and Navβ4 is highly correlated in medium-large diameter neurons, it cannot determine if these subunits interact. All of the neurons co-transfected with Navβ4 and Nav1.6r channels generated fast resurgent sodium currents, supporting the hypothesis that Nav1.6 and Navβ4 interact and suggesting that expression of Navβ4 might be sufficient for fast resurgent sodium current generation in DRG sensory neurons. However, it is important to point out that the neurons typically transfected and recorded from in our sensory neuron expression-system experiments are almost always small diameter sensory neurons; larger diameter neurons do not survive the culturing and transfection procedure very well. It is possible, and even likely, that in some neuronal populations there are other factors that limit the ability of Navβ4 to generate resurgent sodium currents [65].

Navβ2 co-expressed with Nav1.6r did not result in increased resurgent sodium currents. Although the percentage of transfected Navβ2 cells that generated fast resurgent sodium currents seemed slightly lower than that of control neurons, this difference was not significant (nor was the resurgent current amplitude significantly different). Both Navβ4 and Navβ2 are thought to form a disulfide linkage with sodium channel α-subunits and because of this it has been proposed that a given α-subunit may associate with either Navβ4 or Navβ2, but not both. Although Navβ2 overexpression did not significantly reduce fast resurgent current amplitude, this does not necessarily indicate that Navβ4 and Navβ2 can simultaneously associate with α-subunits. One possibility is that Navβ2 may poorly associate with Nav1.6r channels. Another possibility is that the expression of Navβ2 was not high enough to compete with endogenous Navβ4 (although expression was visually confirmed with a fluorescent tag). Interestingly, expression of a mutant Navβ4 also did not reduce the baseline resurgent current amplitude significantly. However, this construct shifted the voltage-dependence of Nav1.6r channel activation and inactivation, indicating it does associate with Nav1.6r channels. This raises the possibility that there might be another open-channel blocker, at least in small diameter sensory neurons, that could contribute to fast resurgent current generation independent of Navβ4 association.

Although Nav1.6 appears to be the predominant generator of fast TTXS resurgent currents in sensory neurons, it is not the only isoform that can generate resurgent sodium currents [15, 32, 66]. Nav1.7 is able to generate TTXS resurgent sodium currents in sensory neurons [15]. Wild-type Nav1.7 is not an efficient generator of resurgent sodium currents [15] despite having similar kinetics of open-state inactivation to Nav1.6 [67], but mutations identified in patients with Paroxysmal Extreme Pain Disorder can substantially increase Nav1.7 resurgent currents [15, 44, 68]. Nav1.8 channels can also generate resurgent sodium currents in DRG sensory neurons [40]. Nav1.8 resurgent currents are much slower and are resistant to TTX. Although we did not examine slow TTXR resurgent currents in this study, it is likely that Navβ4 plays an important role in these currents too as inclusion of a 14-mer peptide corresponding to the proximal portion of the Navβ4 cytoplasmic C-terminus (the first 14 amino acids highlighted in Fig. 6a) significantly enhanced slow TTXR resurgent currents in DRG neurons [40].

Resurgent currents are likely to be an important determinant of sensory neuron excitability. Over-expression of Navβ4 with Nav1.6r substantially enhanced both spontaneous and evoked firing in sensory neurons compared to neurons transfected with Nav1.6r and the fluorescent tag. It is important to note that the activity of endogenous sodium channels were blocked in these experiments, so the increased excitability most likely reflects enhanced Nav1.6r activity. Overexpression of wild-type Navβ4 increased resurgent current amplitude by threefold and shifted the voltage-dependence of inactivation by +4 mV. Based on the small magnitude of the shift in the voltage-dependence of inactivation, we predict that the threefold increase in resurgent currents was the major factor in the increased evoked action potential firing that we observed. Although the mutant Navβ4 did not enhance resurgent currents, it induced a pronounced shift in activation that precluded its use in the current clamp experiments. The increased spontaneous activity observed with wild-type Navβ4 is intriguing, but it is not entirely clear how this might result from enhanced resurgent current activity. Enhanced Nav1.6 resurgent currents have been proposed to underlie the sensory neuronal excitability associated with oxaliplatin and sea anemone toxin ATX-II induced pain sensations [42, 43]. Furthermore, an inflammatory soup applied to cultured DRG neurons increased both TTXS and TTXR resurgent currents, suggesting that resurgent currents can also play a role in inflammatory pain [40]. Inflammatory mediators can increase the activity of multiple kinases and phosphorylation is known to enhance resurgent current generation [68, 69]. It will be interesting to determine if chronic oxaliplatin treatment and/or chronic inflammation can induce an upregulation of Navβ4 in sensory neurons.

## Conclusion

Nav1.6 and Navβ4 expression is highly correlated in medium-large diameter neurons. Navβ4 overexpression enhanced resurgent currents and excitability, whereas knockdown or expression of mutant Navβ4 decreased resurgent current generation. Overall our data demonstrate that Navβ4 is a major contributor to fast resurgent current generation, particularly in larger diameter neurons. Although it is not clear what sensory modalities are most impacted by fast resurgent sodium currents, Navβ4 is likely to be an important determinant of sensory neuronal hyperexcitability and thus could represent an important target for the development of novel therapeutics.

## Methods

### cDNA constructs

These studies used cDNA constructs of sodium channel beta subunits, Navβ2 and Navβ4, which were tagged at the C-terminus to verify expression. To generate rat Navβ4 C-terminal tagged construct, an *Apa*I restriction enzyme site was introduced into pCMV6-Vector before the stop codon (Origene clone, RR210027) using Quickchange XL II Site Directed Mutagenesis kit (Agilent Technologies). The open reading frame for Navβ4 protein (NP_001008880.1) between *Apa*I and *Eco*RI was cut and inserted into mVenus N1 or pmTurquoise2 N1. Vectors, mVenus N1 and pmTurquoise2 N1, were gifts from Dr. Richard Day at Indiana University School of Medicine (Indianapolis, IN, USA). Two constructs were generated using this approach: Navβ4-Turquoise and Navβ4-Venus. A predicted inactive form of Navβ4-tagged (Navβ4-Mt) was generated by converting lysines at position 192–193 and 197–199 to alanine using Quickchange XL II Site Directed Mutagenesis kit. To generate rat Navβ2-tagged construct, cDNA encoding for rat Navβ2 protein (NP_037009.1) was codon-optimized and synthesized. The *SCN2B* open reading frame was cut with *Nhe*I–*Age*I and inserted into mVenus N1 vector.

To generate the Nav1.6r construct, *SCN8A* gene encoding for human Nav1.6 protein (NP_055006.1) was codon-optimized and synthesized. The open reading frame was cut with *Kpn*I–*Xba*I and inserted into pcDNA3.1 vector. The resulting construct was modified by converting tyrosine 371 to serine to confer high resistance to TTX as previously described [63, 67].

To isolate Nav1.6r currents, Nav1.8 was knocked down with Nav1.8 shRNA-IRES-dsRED construct. The Nav1.8 shRNA-IRES-dsRED construct is a vector plasmid (pIRES2-dsRed) that encodes for Nav1.8 shRNA sequence (targeting sequence, GATGAGGTCGCTGCTAAGG [70]) and internal ribosome entry site for the translation of fluorescent protein marker dsRed (IRES-dsRED) as previously described in [15].

### Cell culture

Adult rat DRG ganglia were harvested, dissociated and cultured as previously described in [10, 15, 67]. Briefly, adult male Sprague–Dawley rats were rendered unconscious by CO_2_ exposure and decapitated. The spinal column was removed and dorsal root ganglia were harvested from the lumbar region up to the cervical region. Excised ganglia were digested in Dulbecco’ modified Eagle’s Medium (DMEM, Fisher Scientific) containing collagenase (1.25 mg/mL) and neutral protease (0.78 mg/mL) for 45 min at 37 °C. Ganglia were mechanically triturated with sequentially smaller pasteur pipettes in 10 % Fetal Bovine Serum (FBS, Hyclone) DMEM (Invitrogen). Glass coverslips coated with poly-d-lysine and laminin were loaded with dissociated cell suspension. After 10 min, cells settled and 10 % FBS DMEM was added. For knockdown experiments, L4 and L5 ipsilateral dorsal root ganglia were excised from rats injected with non-targeting control or β4siRNA. The above dissociation protocol and culture was followed with the exception of the digestion time, which was decreased, to 28 min. Cells were maintained at 37 °C in a humidified 95 % air and 5 % CO_2_ incubator. Media was changed every 2 days. For experiment longer than 2 days, such as isolated recordings of Nav1.6r from DRG neurons, 10 % FBS DMEM was supplemented with mitotic inhibitors: 5-fluoro-2-deoxyuridine (50 uM, Sigma Aldrich) and uridine (150 uM, Sigma Aldrich). Indiana University School of Medicine Institutional Animal Care and Use Committee (IUSM IACUC) approved the animal protocols described.

### Procedure for in vivo injection of siRNA into the DRG

siRNA “smartpool” consisting of four different siRNA constructs combined into one reagent directed against rat Navβ4 subunit (Gene ID: 315611) and non-targeting control siRNA were purchased from Dharmacon. Catalog numbers were M-101002-01 (directed against Navβ4) and D-001210-02 (n.t. control, directed against firefly luciferase). The non-targeting control siRNAs are reported to: (1) not target any known rat genes, (2) have a minimum of four mismatches to all human, mouse and rat genes, and (3) have minimal targeting confirmed by genome wide microarray analysis as stated by manufacturer. The four siRNA sequences directed against Navβ4 were: construct 1, GGAUCGUGAAGAAUGAUAA; construct 2, UCCAAGUGGUUGAUAAAUU; construct 3, GCAAUACUCAGGCGAGAUG; construct 4, AAACAACUCUGCUACGAUC. siRNAs were prepared for transfection using cationic linear polyethylenimine-based reagents (in vivo JetPEI, Polyplus Transfection, distributed by VWR Scientific). Aliquots of 3 µL containing siRNA/Jet Pei mixture (80 pmol of siRNA) were injected into each L4 and L5 DRG on one side, through a small glass needle inserted close to the DRG as previously described by [25]. Three days post-injection L4 and L5 dorsal root ganglia were harvested. 16–30 h after culture a fraction of the DRG neurons were examined by immunocytochemistry to verify knockdown and another fraction was used for whole-cell patch clamp (see next sections for more details). IUSM IACUC approved the experimental procedure described.

### Immunocytochemistry

The expression pattern of Nav1.6 and Navβ4 was studied in dissociated cultures of DRG neurons. DRG neurons were fixed after 24 h in culture with 4 % paraformaldehyde (0.1 M phosphate buffer, pH 7.4) for 20 min and washed in phosphate buffered saline (PBS) three times. Cells were permeabilized in 1 % Triton X-100 in PBS for 20 min at room temperature (~22 °C), washed in PBS three times, blocked for 2 h (10 % normal goat serum, 0.1 % Triton X-100 in PBS) at room temperature and washed an additional three times in PBS. Cells were then incubated in primary antibodies diluted in blocking solution overnight at 4 °C. Primary antibodies used were anti-Nav1.6 clone K87A/10 (1:200, AB_2184197, UC Davis/NIH NeuroMab Facility) and polyclonal anti-Navβ4 antibody (1:500, #Ab80539, Abcam). After three washes, cells were incubated with secondary antibodies in blocking solution for 2 h at room temperature. Secondary antibodies used were Alexa Fluor^®^ 488 Goat Anti-Rabbit IgG and Alexa Fluor^®^ 594 Goat Anti-Mouse IgG (Molecular Probes, Life Technologies) at 1:2000 concentration. Coverslips were mounted in Prolong Gold Antifade (Molecular Probes) and DRG neurons imaged using Axio Observer Z1 Widefield Microscope with a 20× objective (ZEISS Microscopy). Images were analyzed using Axio Vision software (Version 4.8.2, ZEISS Microscopy). Each cell was delineated as a region of interest and correlation was determined using the colocalization module of the software. The results recorded for each cell were scatter plot of the two signals, Pearson correlation coefficient and area. The data was grouped into small diameter neurons (<400 μm^2^) and medium-large diameter neurons (>400 μm^2^) and compared using Student’s t test. It is important to note that due to the limitations of this approach, the Pearson correlation values do not specifically represent colocalization of the proteins but rather describe the population of cells that co-express Navβ4 and Nav1.6. Additionally, images were analyzed using NIS Elements Advance Research (Nikon^®^) software and mean intensity for Navβ4 and Nav1.6 staining signal was determined for each cell by defining the region of interest. The data were grouped into small diameter neurons (<400 μm^2^) and medium-large diameter neurons (>400 μm^2^) and compared using Student’s t test.

To verify knock down of Navβ4 protein, L4 and L5 ipsilateral DRG ganglia harvested and cultured from rats injected with non-targeting control and B4-siRNA 3 days after injection were examined. DRG neurons were fixed, permeabilized, blocked and treated with primary anti-Navβ4 antibody and secondary antibody goat Alexa Fluor^®^ 488 Goat Anti-Rabbit IgG (Molecular Probe, Life Technologies); coverslips were then mounted and imaged with Nikon Eclipse TE2000-E confocal microscope with a 20× objective. Corrected total cell fluorescence (CTCF) was calculated in Excel (Microsoft) by applying measurements obtained from NIS Elements Advance Research (Nikon^®^) software using the following equations adapted from [71]:

1 $$ CTCF = Integrated\;density\,-\,(Area\,\,of\,\,selected\,\,cell \,\times\, Mean\,\,fluorescence\,\,of\,\,background\,\,readings) $$

2 $$ Integrated\,\,density = (Mean\,\,intensity\,\,value \times Area\,\,of\,\,the\,\,cell) $$

Quantification experiments were carried out independently at least three times; more than 250 cells were counted for each condition.

### Nav1.6r currents in DRG neurons

The goal of these experiments was to study the modulation of Nav1.6r currents by beta subunits: Navβ4 and Navβ2. Therefore, Nav1.6r currents were isolated and recorded as previously described in [15]. Nav1.6 was made resistant to TTX and endogenous TTXS were blocked with 500 nM TTX. DRG neurons can also endogenously express TTXR sodium currents: Nav1.8 and Nav1.9 [10, 72, 73]. Nav1.8 currents are greatly decreased in culture [15, 74]; additionally Nav1.8 was further decreased by co-transfecting Nav1.8 shRNA-IRES-dsRED construct to minimize contamination Nav1.8 currents in the recordings. Nav1.9 currents are not observed under the culture and recordings conditions [64, 75]. Beta subunits studied, Navβ4-WT, Navβ4-Mt and Navβ2, were tagged at the C-terminus with fluorescent protein (mVenus or pmTurquoise2) to verify expression as described in cDNA constructs section. No difference was observed between modulation of the biophysical properties of human Nav1.6r by co-expression of Navβ4-Turquoise compared to Navβ4-Venus, thus, these construct were used interchangeably in experiments. As a negative control, fluorescent proteins (mVenus or pmTurquoise 2) were co-transfected instead of the beta subunits. No difference was observed between modulation of the biophysical properties of human Nav1.6r by co-expression of pmTurquoise2 compared to mVenus, thus these constructs were used interchangeably in experiments. The Helios Gene Gun (Bio-Rad Laboratories) was used to transiently transfect DRG neurons as described previously described [64, 67, 76, 77]. DRG neurons were co-transfected 36–48 h after dissociation with: Nav1.6r, Nav1.8shRNA-IRES-dsRED, and tagged beta subunit or control (tag only) DNA at a 2:1:1 ratio. DRG neurons that were positive for 1.8shRNA (indicated by dsRed fluorescence) and beta subunit expression (indicated by Turquoise or Venus fluorescence) were selected for whole-cell patch-clamp. In the cells expressing Nav1.6r that were used in the final voltage clamp analysis, the peak recombinant current amplitude averaged 21.2 ± 1.7 nA (n = 94). Cells that expressed Navβ4 or Navβ2 localized only to intracellular compartments were excluded. Cells with residual Nav1.8 current greater than 3 % of the peak current of Nav1.6r were excluded. Nav1.8 contamination can be determined for each individual cell expressing recombinant current by examining the voltage-dependence of steady-state fast inactivation. The midpoint of inactivation for Nav1.8 is much more depolarized compared to Nav1.6. The curve of voltage-dependence of inactivation was used to determine absolute and relative amplitude for Nav1.8 and Nav1.6 [15]. Whole-cell patch-clamp recordings in voltage clamp and current clamp mode were obtained 2–3.5 days after transfection. As an observational note, DRG neurons that were biolistically transfected were generally considered small diameter neurons based on their membrane capacitance (14.9 ± 0.6 pF, n = 123).

### Electrophysiology

Whole-cell patch-clamp recordings were conducted in voltage-clamp or current-clamp mode at room temperature (~22 °C) using a HEKA EPC-10USB. Data were acquired on a Windows-based Intel 2 Core using Patchmaster program (version 2X65; HEKA Elektronik). Fire polished glass electrodes (0.7–1.1 MΩ) were fabricated using a P-97 puller (Sutter), and tips were coated with dental wax to minimize capacitive artifacts and enhance series resistance compensation. The offset potential was zeroed prior to seal formation. Capacitive transients were canceled using computer-controlled circuitry; C-fast for pipette-capacitance correction and C-slow for cell-capacitance compensation. Voltage errors were minimized by series resistance compensation >75 %. Membrane currents were sampled at 20 kHz and filtered online at 10 kHz. Leak currents were linearly cancelled by P/−5 subtraction (pulse/number).

For voltage-clamp recordings, the electrode solution consisted of 140 mM CsF, 10 mM NaCl, 1.1 mM EGTA, and 10 mM HEPES (adjusted to pH 7.3 with CsOH). The extracellular bathing solution contained 130 mM NaCl, 30 mM TEA chloride, 1 mM MgCl2, 3 mM KCl, 1 mM CaCl2, 0.05 mM CdCl2, 10 mM HEPES and 10 mM d-glucose (adjusted pH 7.3 with NaOH). Recording solutions were adjusted using d-glucose to maintain physiological osmolarity values. Whole-cell currents in voltage-clamp mode were not recorded before 4 min after whole cell configuration for Nav1.6r isolation and before 2 min after whole cell configuration for endogenous sodium currents. Cells were held at a potential of −100 mV. I/V relationships were determined by step depolarizations of 50 ms, from −100 to +80 mV, in 5 mV increments. The voltage-dependence of activation (m_∞_) was determined from sodium currents elicited with I/V protocol from holding potential of −100 to 0 mV. Conductance values were calculated at each test potential and normalized to the peak conductance. Data of normalized conductance as a function of voltage was fitted using single-phase Boltzmann distribution from which the midpoint points (V_1/2_) and slope factor (Z) were obtained for each cell. Steady-state fast inactivation (h_∞_) was assayed with 500 ms pre-pulses from −130 to 5 mV (in 5 mV increments) followed by a 20 ms test pulse to −20 mV to assess channel availability. For endogenous current recordings, the fast component of the currents was isolated by pre-pulse subtraction of the slow component as previously described in [78]. Peak currents at each pre-pulse were normalized to the overall peak current. Data of normalized currents as a function of voltage was fitted with single phase Boltzmann distribution from which the midpoint points (V_1/2_) of steady-state fast inactivation and slope factor (Z) were obtained for each cell. Current densities were estimated for each individual recording by dividing the peak transient currents obtained from h_∞_ by the membrane capacitance. Comparison of values for inactivation, activation and current density was done using ANOVA and post hoc Bonferroni test.

For current-clamp recordings, the goal was to study the activity mediated by Nav1.6r under control and Navβ4 over-expression conditions. Thus, transfected Nav1.6r was isolated by silencing the function of other voltage-gated sodium channels with TTX and Nav1.8-shRNA methods as described above. The pipette solution contained 140 mM KCl, 0.5 mM EGTA, 5 mM HEPES and 3 mM Mg-ATP (adjusted pH 7.3 with KOH). The extracellular solution contained 140 mM NaCl, 3 mM KCl, 2 mM MgCl2, 2 mM CaCl2 and 10 mM HEPES (adjusted pH 7.3 with NaOH). Recording solutions were adjusted using d-glucose to maintain physiological osmolarity values. DRG neurons were allowed to settle at their resting potential. Spontaneous activity was defined as continuous firing for 3 or more minutes. DRG neurons that were not spontaneously active were examined for evoked activity with a series of currents injection from −200 pA to 1.2 nA in 100 pA increments. For evoked activity, the maximum number of action potentials elicited from each cell was determined as the maximum action potentials elicited from current injections from 0 to 1.2 nA. For non-spontaneous neurons rheobase was determined as the minimum current injection needed to elicit an action potential response and input resistance (R) was estimated from the membrane potential $(\Delta V)$ change at −200 pA current injection (I) using the equation: V = IR. Comparison of current clamp parameters examined was done using Student t test.

Voltage and current clamp data were analyzed using Origin (version 8, OriginLab), Fitmaster (v2X65, HEKA Electronik), Excel (Microsoft) and Prism (version 6, GraphPad) software programs.

### Resurgent currents and analysis

Cells were assayed with a step protocol that initially depolarized the membrane to +30 mV for 20 ms from the holding potential, followed by repolarizing voltage steps from +15 mV to −85 for 100 ms in −5 mV increments to test for resurgent currents; cells were then returned to their holding potential. Resurgent currents display unique characteristics of slow onset and slow decay along with a non-monotonic I/V relationship. Currents that did not meet these criteria were classified as negative for resurgent currents. Based on these criteria, the percentage of DRG that were positive/negative for detectable resurgent current was quantified for each condition. Chi square test (χ^2^ test) was then used to compare distributions between conditions. Resurgent current amplitudes were measured to the leak-subtracted baseline after 3.0 ms into the repolarizing pulse to avoid contamination from tail currents. Relative resurgent currents were calculated by dividing peak resurgent current by peak transient current and expressed as a percentage of peak transient current. The peak transient current was determined as the peak from the h_∞_ infinity protocol. For endogenous sodium resurgent current recordings, the peak transient of the h_∞_ protocol was determined after subtraction of the slow component using pre-pulse subtraction [78]. For population data, relative resurgent currents were plotted as a function of voltage. Student’s t test was used to examine the statistical significance of relative resurgent current amplitude between groups.

## Additional files

**Additional file 1: Figure S1.** Inactivation properties and peak current of endogenous sodium current in control and Β4siRNA groups. A, Voltage dependence of steady-state fast inactivation is shifted to hyperpolarizing potential in β4siRNA group (brown diamonds, V1/2: 73.55 ± 2.6 mV, slope: 6.601 ± 0.2685 n = 20) relative to non-targeting control (black squares, V1/2: −67.72 ± 2.4 mV, slope: 7.351 ± 0.5701 n = 18). Student t test for inactivation p values: V1/2 = 0.052 and slope = 0.226). B, Total peak current density is not different between non-targeting control (−1.694 ± 0.2123 n = 19) and β4siRNA (−1.434 ± 0.1378 n = 20). C, After pre-pulse subtraction the (TTXS) fast component was isolated from total peak current density. TTXS peak current density was significantly decreased in β4siRNA (1.069 ± 0.1648, n = 20) relative to non-targeting control (1.529 ± 0.1826 n = 19, Student t test: p value < 0.05).
**Additional file 2: Figure S2.** Biophysical properties of Nav1.6r transient current with beta subunit co-expression. Representative traces of Nav1.6r transient current recordings with co-expression of fluorescent tag (control, A, black circles), Navβ4-WT (B, blue squares), Navβ2-WT (C, green triangles) and Navβ4-Mt (D, purple diamonds). E, Navβ4-WT and Navβ4-Mt co-expression significantly shift the voltage dependence of steady-state fast inactivation to depolarizing potentials relative to control whereas Navβ2-WT does not. Navβ4-Mt shifted significantly the voltage dependence of inactivation to more depolarized potentials relative to Navβ4-WT. F, Navβ4-Mt co-expression shifts the voltage dependence of activation of Nav1.6r to depolarized potentials relative to control and Navβ4-WT. Navβ2 and Navβ4-WT do not significantly alter the voltage dependence of activation relative to control. Table 1 contains the values for Boltzmann fit for steady-state fast inactivation, activation and corresponding comparisons.
**Additional file 3: Figure S3.** Navβ4 increased evoked action potentials in response to a range of stimuli intensities. Non-spontaneous cells were stimulated with 1×, 2× and 3× rheobase current injections. Compared to control (n = 15), Navβ4-WT (n = 11) overexpression significantly increased the maximum number of evoked action potential at 1×, 2× and 3× rheobase. Data are mean ± SEM. *p < 0.05, Student’s t test.
