# Supplementary figures and images for: Navβ4 regulates fast resurgent sodium currents and excitability in sensory neurons
Source: Mol Pain. 2015 Sep 25;11:60. doi: 10.1186/s12990-015-0063-9 (PMC4582632; doi:10.1186/s12990-015-0063-9)

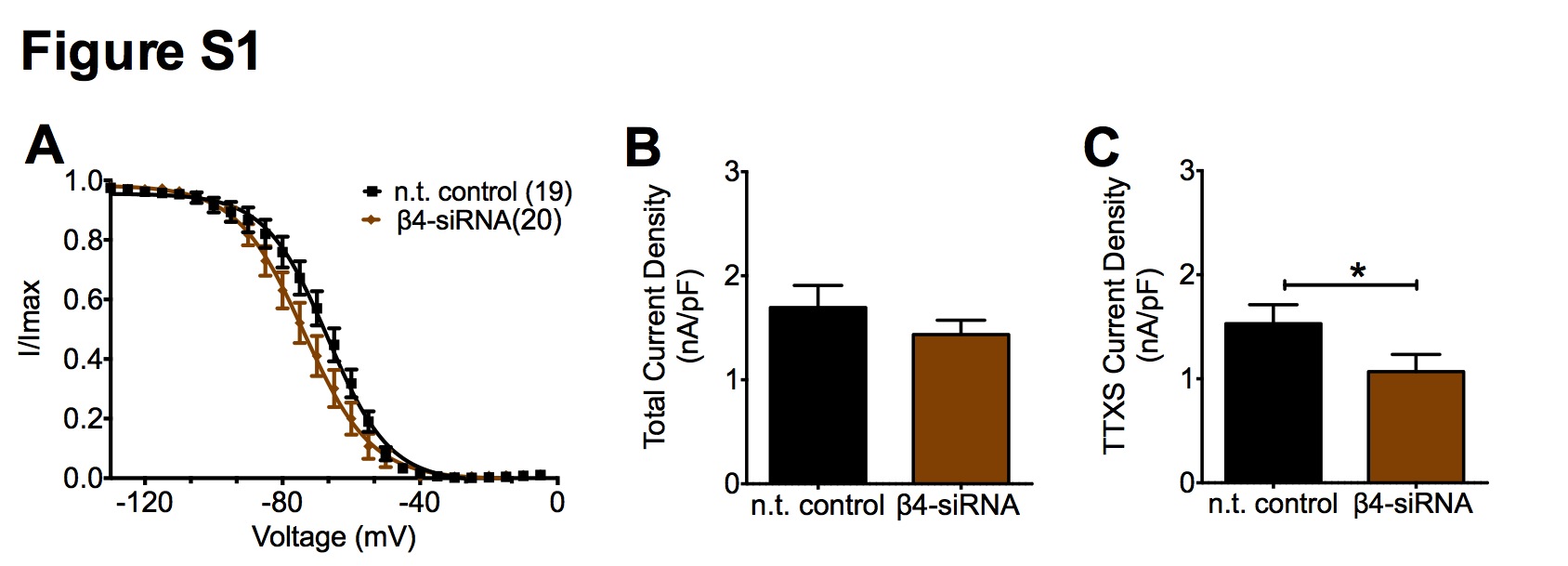

Supplement: Supplementary file 1 — Additional file 1: Figure S1. Inactivation properties and peak current of endogenous sodium current in control and Β4siRNA groups. A, Voltage dependence of steady-state fast inactivation is shifted to hyperpolarizing potential in β4siRNA group (brown diamonds, V1/2: 73.55 ± 2.6 mV, slope: 6.601 ± 0.2685 n = 20) relative to non-targeting control (black squares, V1/2: −67.72 ± 2.4 mV, slope: 7.351 ± 0.5701 n = 18). Student t test for inactivation p values: V1/2 = 0.052 and slope = 0.226). B, Total peak current density is not different between non-targeting control (−1.694 ± 0.2123 n = 19) and β4siRNA (−1.434 ± 0.1378 n = 20). C, After pre-pulse subtraction the (TTXS) fast component was isolated from total peak current density. TTXS peak current density was significantly decreased in β4siRNA (1.069 ± 0.1648, n = 20) relative to non-targeting control (1.529 ± 0.1826 n = 19, Student t test: p value < 0.05). [file 12990_2015_63_MOESM1_ESM.jpg]

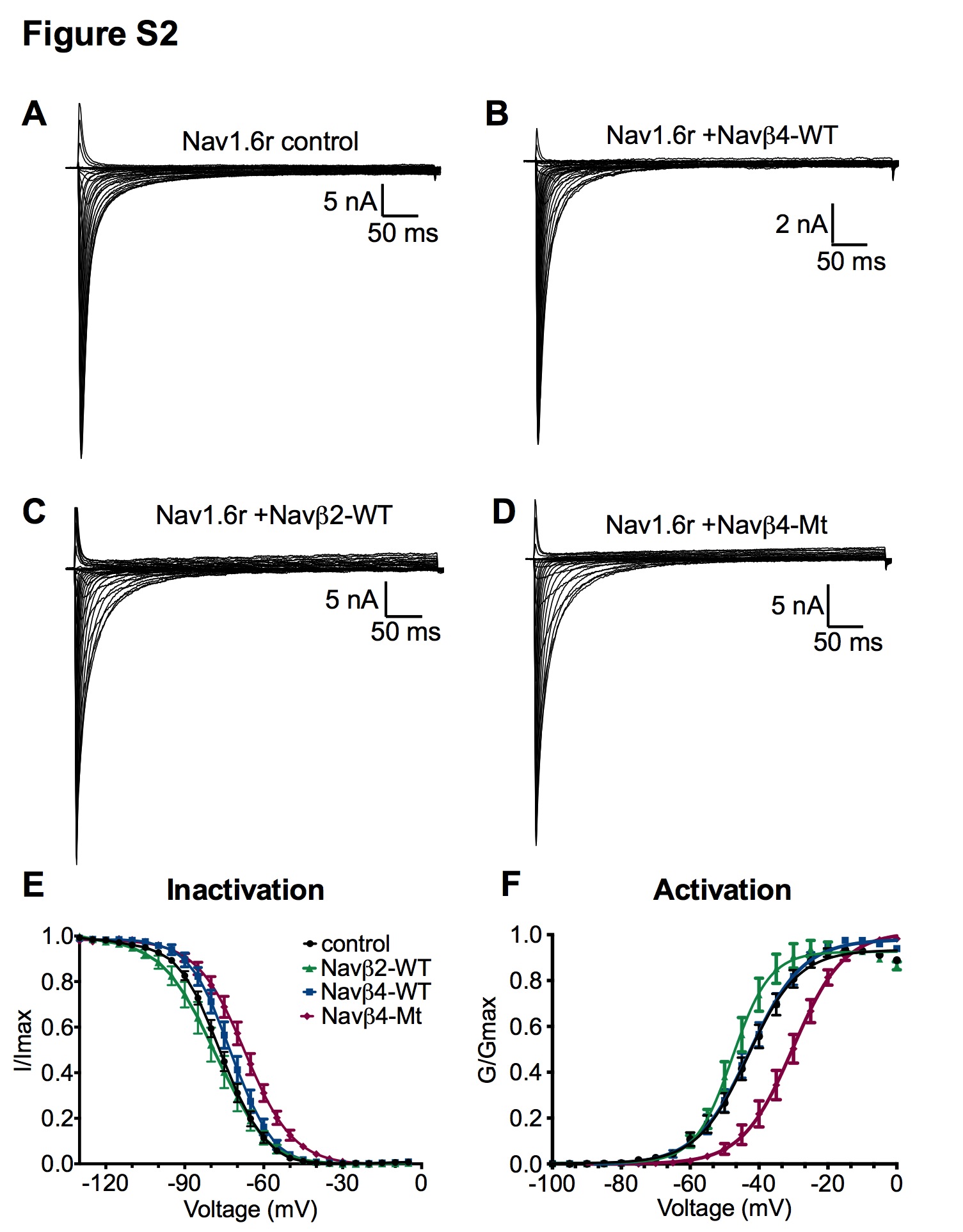

Supplement: Supplementary file 2 — Additional file 2: Figure S2. Biophysical properties of Nav1.6r transient current with beta subunit co-expression. Representative traces of Nav1.6r transient current recordings with co-expression of fluorescent tag (control, A, black circles), Navβ4-WT (B, blue squares), Navβ2-WT (C, green triangles) and Navβ4-Mt (D, purple diamonds). E, Navβ4-WT and Navβ4-Mt co-expression significantly shift the voltage dependence of steady-state fast inactivation to depolarizing potentials relative to control whereas Navβ2-WT does not. Navβ4-Mt shifted significantly the voltage dependence of inactivation to more depolarized potentials relative to Navβ4-WT. F, Navβ4-Mt co-expression shifts the voltage dependence of activation of Nav1.6r to depolarized potentials relative to control and Navβ4-WT. Navβ2 and Navβ4-WT do not significantly alter the voltage dependence of activation relative to control. Table 1 contains the values for Boltzmann fit for steady-state fast inactivation, activation and corresponding comparisons. [file 12990_2015_63_MOESM2_ESM.jpg]

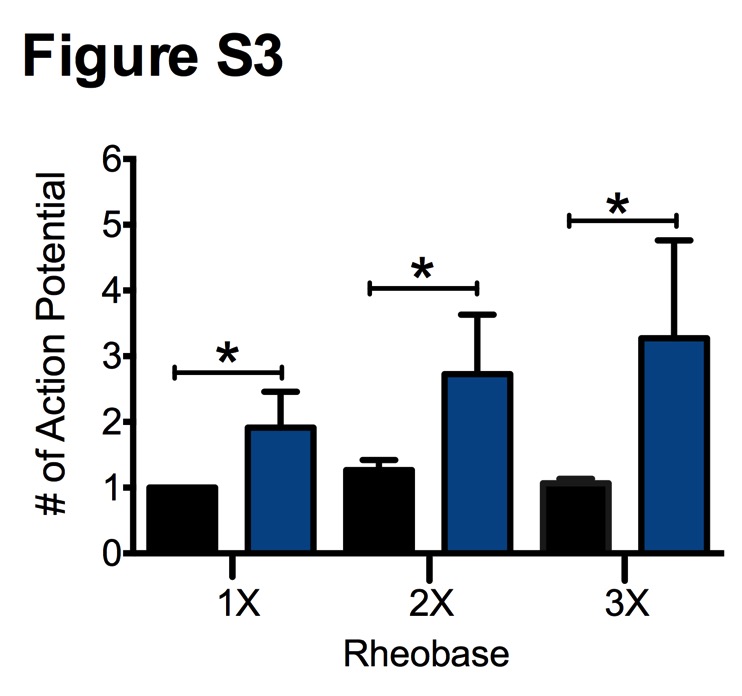

Supplement: Supplementary file 3 — Additional file 3: Figure S3. Navβ4 increased evoked action potentials in response to a range of stimuli intensities. Non-spontaneous cells were stimulated with 1×, 2× and 3× rheobase current injections. Compared to control (n = 15), Navβ4-WT (n = 11) overexpression significantly increased the maximum number of evoked action potential at 1×, 2× and 3× rheobase. Data are mean ± SEM. *p < 0.05, Student’s t test. [file 12990_2015_63_MOESM3_ESM.jpg]
